# Mechanical Properties and Water Permeability of Textile-Reinforced Reactive Powder Concrete with Lightweight Aggregate

**DOI:** 10.3390/ma16247619

**Published:** 2023-12-12

**Authors:** Marcin Różycki, Izabela Hager, Tomasz Zdeb, Mateusz Sitarz, Katarzyna Mróz, Jarosław Zdeb, Natalia Smorońska

**Affiliations:** 1 Footprint—Student Research Club of Buildings Materials Engineering, Cracow University of Technology, 31-155 Cracow, Poland; marcin.rozycki@student.pk.edu.pl (M.R.); natalia.smoronska@student.pk.edu.pl (N.S.); 2 Chair of Building Materials Engineering, Faculty of Civil Engineering, Cracow University of Technology, 31-155 Cracow, Poland; izabela.hager@pk.edu.pl (I.H.); katarzyna.mroz@pk.edu.pl (K.M.); 3 Synergy Building Structures Consortium, 30-605 Cracow, Poland; jzdeb@synergia-kb.pl

**Keywords:** RPC, TRC, textile reinforcement, fibre reinforcement, cement composites, thin-wall structures, modelling

## Abstract

This paper focuses on the development of thin-walled panels with specific properties for applications such as water-tight structures. The authors propose the use of textile-reinforced concrete (TRC) as a composite material and highlight its advantages, which include high tensile strength, improved crack resistance, and design flexibility. The study presents a novel approach which combines TRC with reactive powder concrete (RPC) as a matrix and a lightweight aggregate. RPC, known for its brittle behaviour, is reinforced with glass fibres and a textile fabric to increase its flexural strength. The research includes a comprehensive analysis of the physical and mechanical properties of both the unreinforced RPC matrix and the TRC composite. In particular, the lightweight aggregate RPC matrix has a porosity of 41%, and its mechanical properties, such as flexural and compressive strength, are discussed. The TRC composites, produced in thicknesses ranging from 1 mm to 4 mm, are subjected to flexural tests to evaluate their behaviour under load. The thicker elements show typical damage phases, while the thinner elements show greater flexibility and elasticity. SEM observations confirm good adhesion between the glass fibres and the RPC matrix. Water permeability tests show that the TRC composite, despite its highly porous structure, achieves a water permeability two orders of magnitude higher than that of a reference material, highlighting the roles of both the porous aggregate and the matrix hydration. The paper concludes with a proof of concept—a canoe called the PKanoe, which is constructed from the developed TRC composite. The design of the canoe is supported by numerical analysis to ensure its optimal shape and structural integrity under load. The research contributes to the exploration of innovative materials for sustainable civil engineering applications and addresses both structural and environmental considerations.

## 1. Introduction

Building materials engineering deals with the development of innovative material solutions for civil engineering applications. To design the material that presents new tailored properties, the target purpose of the material is of high importance. However, nowadays, its sustainability and environmental impact must also be considered. The global demand for habitable and agricultural land is increasing, reducing the earth’s natural landscapes. Thus, exploring strategies to integrate water surfaces for cultivation and habitation appears justifiable. Material and structural solutions that enable the development and construction of artificial islands or floating platforms deserve attention and should be further explored. In this research, the authors consider the problem of developing thin-walled panels that present their lightness, resistance to brittle failure, and impermeability to water. Such material is to be used as a composite for the production of a thin-walled shell structure that can float on the water. An example of such an exploration is a mineral composite made of textile-reinforced concrete (TRC) [1]. Moreover, to improve the tightness and mechanical properties, it seems reasonable to apply an ultra-high-performance mineral matrix of reactive powder concrete (RPC) [2,3].

TRC is a material in which non-metallic reinforcement in the form of a textile mesh is placed in a mineral matrix [4]. Textile reinforcement can be easily incorporated into precast thin-wall elements, allowing for faster and more efficient construction [5]. TRC offers several benefits, such as high tensile strength, improved crack resistance, lower weight, and greater design flexibility, especially compared to ordinary reinforced concrete [6]. Moreover, studies [7] show that TRC has good resistance to freeze–thaw erosion. TRC also has better thermomechanical performance against fire than traditional reinforced concrete [8,9].

Research studies involving the TRC assessment are carried out worldwide [10,11,12], and innovative material solutions are being implemented in civil engineering, e.g., as a repairing component [13] and as the composites of ultra-high mechanical properties [14]. In recent years, the scope of TRC research has expanded to include various applications, including strengthening applications, façade panel solutions, or shell structures [15,16,17]. This composite material enables the fabrication of thin-walled concrete elements that provide possibilities for the making of designs of more complex architectural forms. With TRC, it is possible to achieve high-quality surfaces with sharp or rounded edges. Thanks to the corrosion resistance of the textile fibres and yarns that create a textile mesh, the thickness of the concrete cover can be significantly reduced. Thus, it is possible to produce slender structural elements with a wall thickness of 10 mm or less [18]. Textile reinforcement is characterised by high mechanical properties; for example, the tensile strength of glass textile reinforcement reaches 2 GPa [19], while for carbon textile reinforcement it exceeds 4 GPa [20], which is up to eight times higher than the tensile strength of steel reinforcement. Reducing the thickness of the structural element leads to a reduction in the amount of material used and thus limits the carbon footprint of the final product—thin-walled composite panels [21].

Concrete is a structural material whose performance is significantly affected by insufficient tensile strength [22] and thus it is prone to e.g., extensive cracking. Adding reinforcement to the concrete matrix is an effective method for improving its ductility and tensile strength. For fibre-reinforced concrete (FRC), glass [23], carbon [24], or basalt fibres [25] are mostly used. The raw materials forming the textile reinforcement and fibre reinforcement are the same, but the textile reinforcement is located in the element cross-section in the areas located exactly where the tensile stresses are designed to occur. The properties of textile reinforcement can be fully exploited as it is placed in the desired position in a sufficient quantity. Conversely, the fibres are randomly dispersed and oriented through the cross-section of the element [26]. In general, more than 3% of the short fibres by volume is required to effectively reinforce concrete products [27]. Therefore, TRC can reduce the cost of structures compared to FRC by introducing a lower amount of fibres [24]. A lot of data can be found in the literature regarding the effect of various reinforcement parameters, such as the type, density, and diameter of the yarn, as well as the pattern and structure of the textile weave, on the properties of textile-reinforced concretes [28]. Depending on the type of textiles in the composites, the following types of TRC can be distinguished:

- CTRC—carbon textile reinforced concrete [29];
- GTRC—glass textile reinforced concrete [30];
- BTRC—basalt textile reinforced concrete [31];
- and others [32].

The flexural strength of the composite increases with greater yarn density and the number of fabric layers. In [33], it has been proven that the higher density of the cotton textile improves the failure characteristics of TRC concrete. The presence of the textile reinforcement in concrete delayed the appearance of the first crack, and the tensile stress leading to the first crack was doubled between the composites with one- and four-layers of textile reinforcement (2.1 MPa and 4.1 MPa, respectively). Also, the increase in number of the cotton textile layers increased the tensile strength (3.5 MPa to over 7 MPa for cotton fabric cement composites). As reported in [34], by increasing the number of textile layers in the part of the element working in tension, the mechanical properties of TRC are improved.

To increase the mechanical strength of the composite, especially in terms of its resistance to shear stresses, it was found to be effective to use fibres in combination with textile reinforcement. Such cooperation can prevent interlayer shear phenomena, which results in the more effective work of the reinforcement. Test results [35] show that the load-bearing capacity can be increased by up to twice that of conventional TRC.

In addition to the development of TRC and FRC composites, the strength of the mineral matrix should also continue to improve. If the mechanical properties of the matrix do not match the high performances of textile reinforcement, it can lead to interlayer shear failure, significantly reducing the performance of the textile-reinforced composite [35].

The cement matrix for TRC composite production must meet the relevant requirements in terms of both technical and mechanical properties [36]. The composition of the mixture must ensure the right consistency for the chosen moulding method and, after hardening, provide adequate tightness, strength, and durability. To ensure good interfacial transition zone properties, the fresh matrix should present good workability while the hardened materials should be tight and impermeable. To ensure the high mechanical properties of a matrix, a high strength class of CEM I 52.5 cements are usually used. Moreover, as the water to cement ratio shall stay low, the highly effective superplasticisers sre required. In addition, a fine-grained concrete matrix made with a large amount of powders (quartz, lime, and microsilica) guarantees an even and smooth surface right after formwork disassembly. Summing up the requirements given for the mineral matrix, the reactive powder concrete [2,3] seems to provide a satisfactory solution which can achieve tailored results.

This paper presents the possibility of producing thin-walled cement composites on a lightweight aggregate with a matrix of reactive powder concrete. Such a fusion of an ultra-high performance RPC matrix with a lightweight aggregate and both textile and fibre reinforcement has not been presented before in the literature. Moreover, the work of fracture and the water permeability determination of TRC are hardly found in the literature. The paper also explains the pseudo-plastic behaviour of the brittle mineral RPC matrix under bending conditions. In this experimental campaign, textiles in the form of a glass fibre mesh with the addition of dispersed glass fibres are used as a reinforcement. The research carried out concerns the determination of the properties of the unreinforced mineral RPC matrix—density, total porosity, and flexural and compressive strength. The thin-wall shells are manufactured with textile and fibre-reinforced RPC with the different thicknesses of 1 mm, 2 mm, 3 mm, and 4 mm to achieve the minimum thickness with the tailored mechanical properties. The behaviour of the composite under three-point bending is presented. The work of damage under bending is determined based on the force–deflection relationship. Moreover, water permeability tests are carried out using the GWT method (German Water Permeation Test).

Based on the experimental evaluation of the reinforced RPC, the proof of concept is demonstrated by the construction of a canoe using the developed composite material. To design the required textile reinforcement and the proper shape of the canoe, a numerical analysis in terms of its displacement, deformation, and stress concentration under the anticipated load is performed.

**Figure 1:**
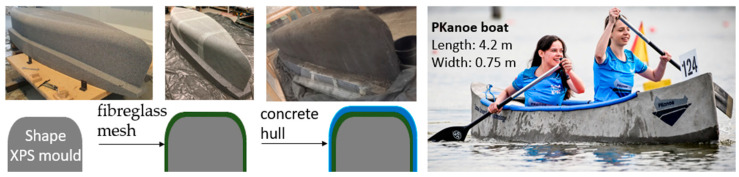
PKanoe boat—production technology (Archives of the FOOTPRINT Student Research Club).

The construction of the canoe with textile-reinforced RPC was performed by the “Footprint” Student Scientific Group from the Cracow University of Technology. The composite used to manufacture the structure was adopted from the experimental studies. The canoe, named PKanoe, took part in the 18th Deutsche Betonkanu Regatta. This proved that the assumed criteria for the developed composite were met and that the shape of the canoe was properly designed. The PKanoe manufacturing stages and its use are presented in Figure 1.

## 2. Materials and Methods

### 2.1. Characteristics of Reactive Powder Concrete Components and Reinforcement

**Figure 2:**
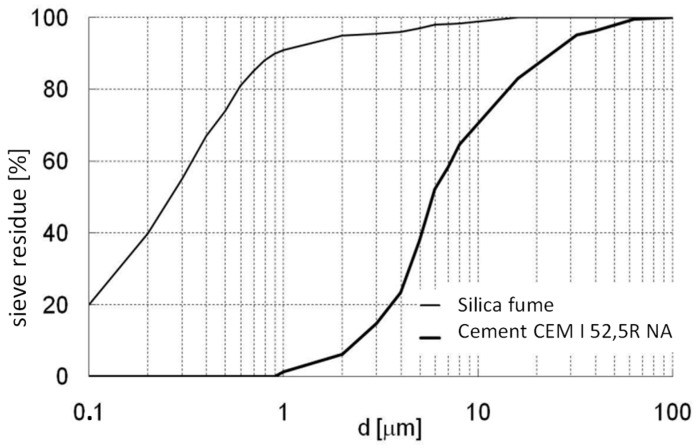
Particle size curves of Silimic silica fume and CEM I 52.5R NA cement.

The CEM I 52.5 CHORULA Górażdże, Poland Portland cement was used to prepare the RPC (reactive powder concrete) mix. Detailed information on the properties and composition of the cement is provided in Table 1, Table 2 and Table 3. The particle size distribution is presented in Figure 2.

Silica fume, which is available under the trade name Silimic (Figure 2) and is obtained from the production of iron–silicon alloys at the Łaziska steel mill, was also used. The grain size distribution is shown in Figure 2, and the raw material characteristics are shown in Table 4.

**Figure 3:**
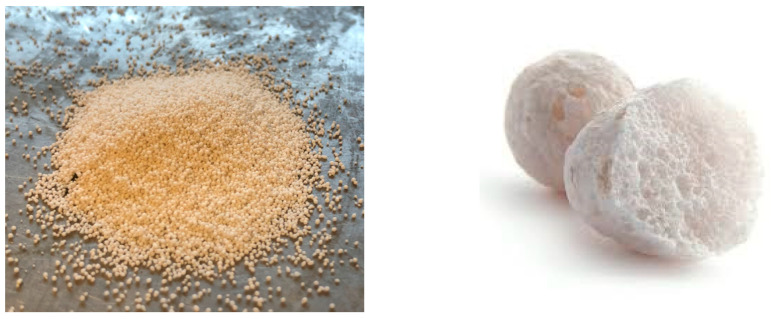
Lightweight aggregate “Stikloporas” and its porous, foamed internal structure [37].

The quartz meal used to prepare the mixture came from the OSIECZNICA Sp. z o.o. Glass Sands Mine and Processing Plant. In addition, the fine lightweight aggregate of expanded glass—Stikloporas Lithuania—was used (Figure 3). It is an inorganic thermal insulation material made from recycled glass, in the form of small porous granules. Two fractions were used: LA fine 0–0.25 mm and LA coarse 0.25–0.5 mm. The chemical composition, the basic physical parameters, and the grain size characteristics are presented in Table 5 and Table 6.

Tap water, as specified in EN 1008, and SIKA ViscoCrete 20 Gold polycarboxylate superplasticiser were used for the concrete mixture. The admixture made it possible to significantly reduce the amount of mixing water up to a water/binder ratio of 0.2.

The textile reinforcement of the composite was a glass fibre mesh with a basis weight of 110 g/m^2^. The fibre, made of alkali-resistant zirconia glass and Cem-Fil AR Anticrack HD, manufactured by Saint-Gobain RF, was also used as an additional dispersed reinforcement. The basic properties of the reinforcement are shown in Table 7.

### 2.2. Composition and Specimen Preparation

The mix was prepared according to the previous research results of a study of reactive powder concrete carried out by T. Zdeb [2,3]. The composition of the prepared mixture is presented in Table 8. The mixing process was carried out in a HOBART HSM 20 mixer equipped with a 20 dm^3^ bowl. The agitator, performing a planetary motion, rotated around its axis at a frequency of 110 rpm. The prepared mixture was used to make small beam samples of 40 mm × 40 mm × 160 mm and plates of four thicknesses: 1 mm, 2 mm, 3 mm, and 4 mm manufactured in moulds of 0.3 m × 0.3 m.

All the samples made were kept under foil for 7 days to prevent the evaporation of water and then under laboratory conditions of 18 °C and RH 75% until testing.

### 2.3. Test Methods for Selected Properties of RPC and TRC Composite

The bulk density of the RPC material was determined by direct measurement of the geometry and the mass of samples dried to a constant weight at 105 °C. Specific density was determined using a helium pycnometer (Quantachrome Ultrapyc 1200e). The porosity of the material was estimated using the relationship between bulk density and specific density, as given in Formula (1).

(1) $$P=\frac{\rho -\rho_{b}}{\rho }\cdot 100\%$$

where

ρ−specificdensity
$\rho_{b}-bulkdensity$

The porosity of the composite was tested by mercury intrusion porosimetry (MIP) with a Quantachrome Poremaster 60. The samples for porosity testing were cut from 40 mm × 40 mm × 160 mm beams using a table saw under wet conditions. After cutting, the pieces had a cross-section of 1 cm^2^ and a height of approximately 3 cm. The materials were then cleaned under tap water and dried.

**Figure 4:**
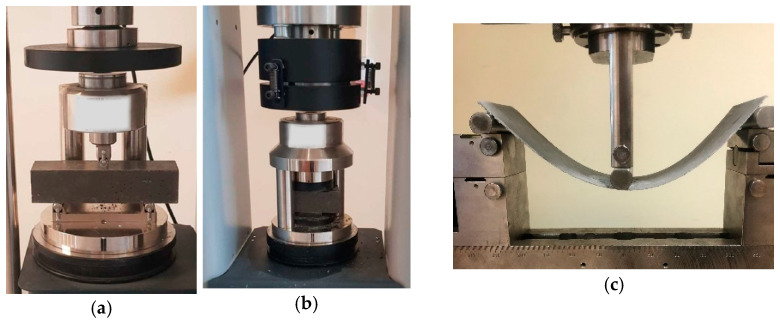
(**a**) Flexural tensile strength test of the RPC matrix; (**b**) compressive strength test of the RPC matrix; (**c**) bending of the textile-reinforced composite, 1 mm thick.

The flexural tensile strength of the RPC mortar was determined on three 40 mm × 40 mm × 160 mm samples after 28 days using the CONTROLS testing machine. The test was performed following the standard PN-EN 196-1:2016 [38] for mortar testing; see Figure 4a.

The compressive strength test of the RPC mortar was performed on the fragments remaining after the flexural tensile strength test, according to the standard EN 196-1:2016: “Methods of testing cement—Part 1: Determination of strength”; see Figure 4b.

The bending strength was also determined for the manufactured TRC composites. The tests were carried out on a Zwick-Roell Z50 testing machine. The 3-point static bending tests were performed on 300 mm × 100 mm thin-walled plates with thicknesses of 1 mm, 2 mm, 3 mm, and 4 mm that were cut from 300 mm × 300 mm boards of the respective thickness. The spacing between the supports was 240 mm. For each composite thickness, the test was performed after 7 and 28 days; see Figure 4c.

During the bending test of the TRC plates, the deformation of the specimen was also recorded. Due to the expected large deformation of the composites, it was decided to measure the deflection by moving the crosshead; this was recorded to an accuracy of 0.001 mm. The tests were carried out up to a deflection of 40 mm. For this range, the work of damage of the composites was determined, unless the material had previously failed. Measurements were made at a constant traverse displacement of 1 mm/min. The measured displacements were adopted to calculate the total damage energy. By this approach, it was possible to determine the residual stresses and to calculate the total work of damage. The value of the work of damage U was determined using Formula (2) following the first law of thermodynamics, assuming that no heat transfer took place during the deformation process.

(2) $$U=U_{e}+U_{d}$$

where U_e_ means the elastic strain energy, while U_d_ is the dissipation energy, i.e., the energy consumed during the internal damage and plastic deformation of the specimen. The sum of these two energies corresponding to the work of damage was determined from Equation (3).

(3) $$U=\int_{0}^{\delta_{n}}F_{i}{d\delta }_{i}=\sum_{i=0}^{i=n}\frac{1}{2}(F_{i}+F_{i+1})(\delta_{i+1}-\delta_{i})$$

The microstructural tests, which included observation of the contact zone between the paste and glass fibre, as well as the compatibility between the paste and textile reinforcement, were conducted using a Zeiss (Oberkochen, Germany) EVO-MA 10 scanning electron microscope (SEM). The samples for SEM observation were cut from the plates after the bending strength test. Any special methods for sample preparation before SEM analysis were not applied.

**Figure 5:**
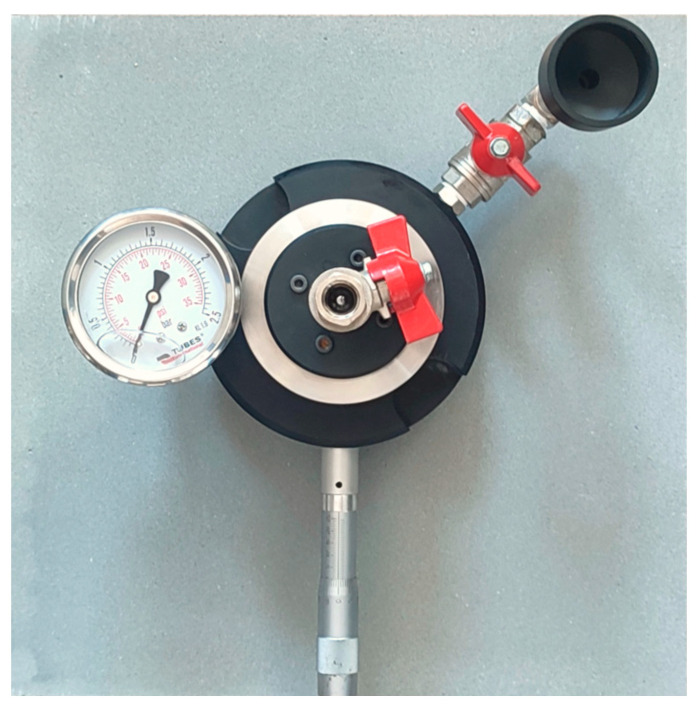
Water permeability measuring device on 4 mm TRC plate.

The water permeability of the TRC composite was tested according to a method developed by Germann Instruments using the Germann Water Permeability Testing Device GWT [39]. The German Water Permeation Test works on the principle of the quantification of water infiltration into a block of concrete under controlled pressure conditions. To achieve this, a pressure chamber fitted with a watertight seal is fixed to the surface. Once the chamber is filled with water and the filling valve is closed, the top cap of the chamber is turned until the target level water pressure. In the literature, the target pressure level is 100 kPa. As the water seeps into the concrete, a micrometre gauge is used to apply force to a piston inside the chamber to maintain the pressure of 100 kPa. The movement of the piston compensates for the volume of water that penetrates the material. The displacement of the piston over time is used to characterise the permeation of the surface being tested. The experimental device for the GWT test is presented in Figure 5.

The following equation is used to determine the water flow rate q:

(4) $$q=\frac{B(g_{1}-g_{2})}{A\cdot t}$$

where

q—penetrating water flow (mm/s);
B—core diameter of a micrometre screw screwed into the measuring chamber to maintain a constant pressure while measuring (mm^2^);
g_1_, g_2_—the position of the micrometre core before (1) and after testing (2) (mm);
A—surface area of water penetration into the test specimen (mm^2^);
t—measurement time (s).

The experimental tests were carried out on three samples; when analysing the results obtained, the average value was most often taken into account. If the single result deviated from the average value by more than ±15%, it was rejected, and the test was repeated until homogeneous results were obtained.

## 3. Results and Discussion

### 3.1. RPC Matrix

#### 3.1.1. Physical Properties of the RPC Matrix

The use of a lightweight aggregate in the RPC significantly reduced the density of the material. A bulk density for the RPC mortar of 1.23 g/cm^3^ was obtained. At the same time, the density measurement in the helium pycnometer was 2.08 g/cm^3^. Using both bulk and specific density values and Equation (1), a porosity value of 41% was calculated. Table 9 shows the values of the determined characteristics.

#### 3.1.2. Porosity of RPC Matrix

**Figure 6:**
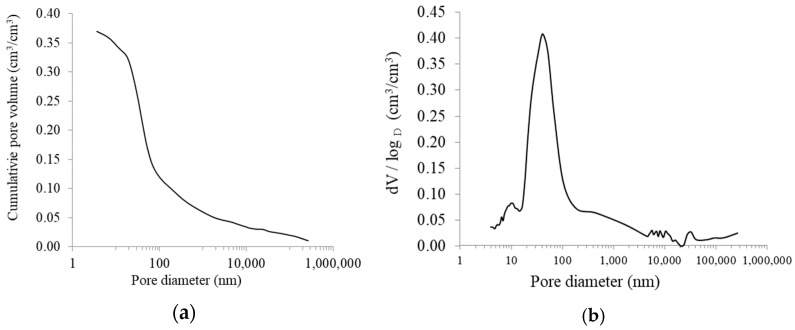
Porosity of RPC matrix with lightweight aggregate: (**a**) total porosity, (**b**) pore size distribution.

In addition to the porosity determined from the density relationship, a more detailed analysis was carried out using mercury porosimetry. This method allowed the total porosity value to be estimated and the pore size distribution to be analysed. This extended analysis provided a better overview of the composite porosity. Porosity was tested 28 days after material manufacture. Figure 6 shows the results, which are the average of the three measurements.

The porosity reached values of 0.37 cm^3^/cm^3^. This represents a total porosity of 37%. The value obtained (37%) is lower than the value (41%) obtained from the density relationship. This discrepancy is correct and is due to the limitations of the mercury porosimetry method. Only open porosity can be measured and only within a certain range of pore sizes. The pore size distribution is shown in Figure 6b. By analysing the distribution curve, it can be seen that the largest number of pores present in the material is between 0.02 and 0.08 μm. The majority of the pores are 40 nm in size, which can be seen as a peak on the graph. The shares of the porosity in a particular range of pore diameters are listed in Table 10.

**Figure 7:**
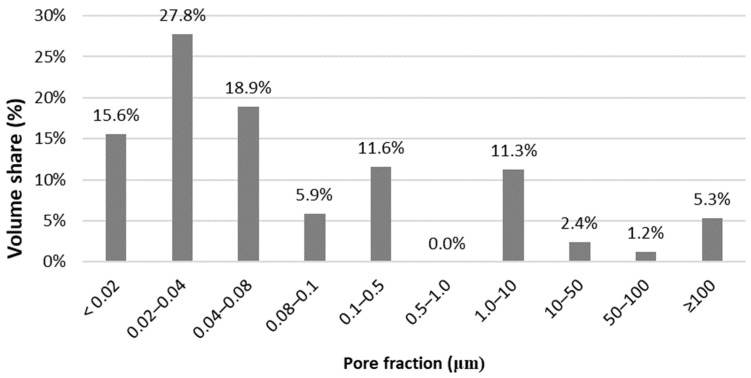
Percentage share of each pore fraction in the RPC matrix.

Based on the data from Table 10, a bar chart was prepared which shows the percentage of each fraction in the open porosity of the material; see Figure 7. The structure of the material is dominated by pores with small diameters. More than 60% of the pores are in the range of 0.02 to 0.08 μm, with the largest group being 0.02–0.04 μm, which accounts for almost 30% of the total measured porosity.

#### 3.1.3. Mechanical Properties of the RPC Matrix

The average flexural tensile strength at 28 days of the matrix was 12.0 MPa. For compressive strength, the average value reached 59 MPa. Due to the use of a lightweight aggregate with a porosity of 85%, the compressive strength was reduced by more than three times compared to a material based on quartz aggregate that was presented in the study in [21]. To be highlighted is the fact that the ultra-high-performance cement matrix is known for its brittle behaviour. In general, the ratio of flexural tensile strength to compressive strength has an average of 5%. This compares favourably with an average of 10% for plain concrete. In the presented case, the f_f_/f_c_ ratio for the RPC matrix was as high as 20%. This is also reflected further in the work of damage results for the RPC composite reinforced with textile and fibre reinforcement.

### 3.2. Textile and Fibre-Reinforced RPC Composite

#### 3.2.1. Mechanical Properties of the TRC

**Figure 8:**
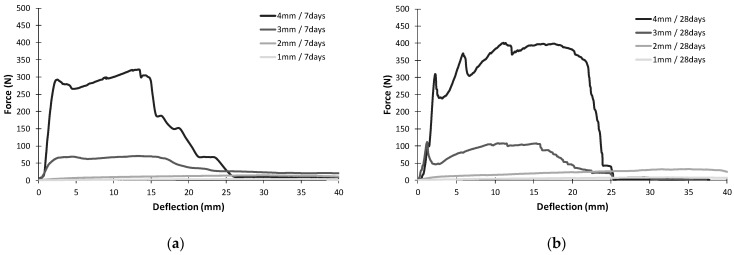
Relationship between bending force and deflection for samples of different thicknesses: (**a**) after 7 days of curing, (**b**) after 28 days of curing.

Figure 8 shows the representative relationship between the bending force (N) and the plate’s deflection (mm), as determined during a static three-point bending test. The tests were carried out on the specimens aged 7 and 28 days. The tests were carried out for the plates with thicknesses of 1 mm, 2 mm, 3 mm, and 4 mm, as described in the previous paragraph.

**Figure 9:**
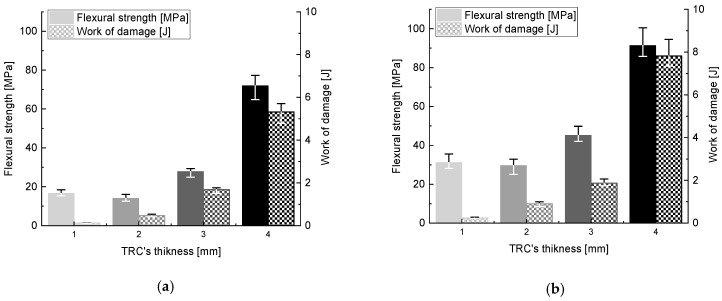
Flexural tensile strength and work of damage: (**a**) after 7 days of curing, (**b**) after 28 days of curing.

In comparing the failure behaviour of the composites, two trends can be observed. In the case of the plates with thicknesses of 3 mm and 4 mm, three phases typical for fibre-and textile-reinforced concretes can be identified in the graphs: Phase I—the elastic behaviour of the cement matrix until the first crack; Phase II—the load transferred by reinforcement and the formation of cracks; Phase III—the cracking of the textile fibres. The thinner elements (1 mm and 2 mm) do not have typical damage phases. These elements are more flexible and elastic. The samples with thicknesses of 1 mm and 2 mm fail at a significantly lower force but are capable of larger deformations. During the tests, it was possible to record displacements of more than 40 mm. The specimens kept their continuity at high bending due to the reinforcement. The specimens of 3 mm and 4 mm thick were able to carry considerably higher forces. However, after matrix cracking, the extent of the displacement observed was smaller. After 7 and 28 days, the specimens failed at a deflection of approximately 25 mm. The failure rate at the age of 28 days was more rapid. The differences in the thickness-dependent behaviour of the composites may be due to the different ratio of contribution of the reinforcement in cross-section. The cross-sections of the thin elements were filled with reinforcement to a greater degree when compared to the 3 mm and 4 mm thick elements. This may be justified by the lower volume fraction of the mineral matrix, which reduces the stiffness of the element and allows greater deformation. The observed differences in bending behaviour are also reflected in the work of damage presented in Figure 9.

The value of the work of damage increases with the thickness of the composite. Differences were visible after both 7 and 28 days of curing. For the composites of smaller thicknesses, 1 mm and 2 mm, after 28 days the increase in the work required for damage was double compared to the value calculated after 7 days of curing. For the 3 and 4 mm thick composites, the increases were considerably smaller, and between 7 and 28 days of curing, they were about 10% and 45%, respectively. Regardless of the curing time, a significant rise in flexural tensile strength, calculated by taking into account the maximum force recorded during the test, was observed as the thickness of the test specimen increased. After 28 days of maturing, the strength was about three times higher, reaching a value of approximately 90 MPa. This enormous variation in results is due to the structure of the TRC, where, in the case of the thin slabs, the position of the reinforcement mesh practically coincided with the neutral axis of the specimen; in the case of the thicker slabs, once they were cracked, the tensile stresses were taken over by the well-anchored mesh, while the high-strength RPC composite took over the compressive stresses in the upper region of the bending element. Figure 8 shows the average flexural tensile strengths and work of damage, with the minimum and maximum values obtained in a series of three specimens.

#### 3.2.2. SEM Observations of TRC

**Figure 10:**
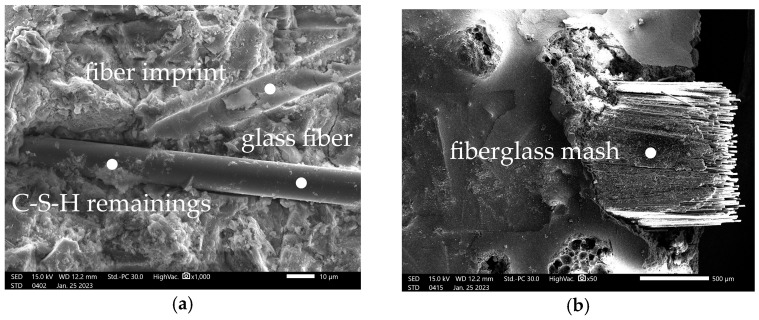
SEM observations: (**a**) concrete structure with Cem-Fil AR Anticrack HD fibres (×1000), (**b**) textile with visible fibrous structure (×50).

**Figure 11:**
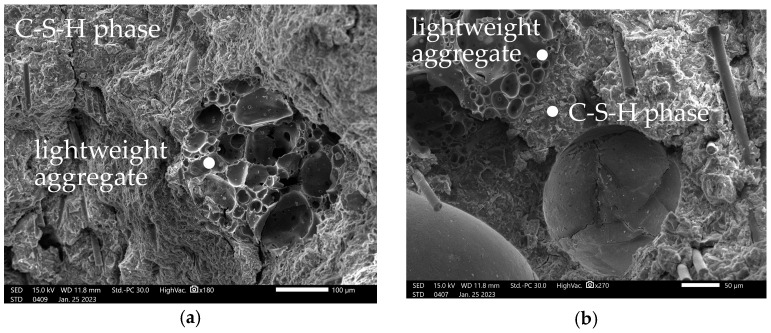
SEM observations: (**a**) cement matrix structure and interface between Stikloporas porous aggregate and RPC matrix (×180), (**b**) RPC composite structure with fibres (×270).

Figure 10a,b present the microstructure of RPC with reinforcement, i.e., with Cem-Fil AR Anticrack glass fibres and the yarn forming a textile mesh with a visible fibrous structure, while Figure 11a,b show the contact zone of the cement matrix and the lightweight aggregate of Stikloporas expanded glass with an expanded porous structure, as well as the porosity of the observed composite.

Figure 10a confirms the good adhesion of the glass fibres to the RPC matrix, i.e., mainly to the C-S-H phase. The residues of hydrated calcium silicates can easily be observed on the surface of a single fibre. In addition, a damaged matrix structure (partial cohesion breakdown) can be seen in the space around the imprint created when the fibre is pulled out, indicating that the matrix is highly adherent to the fibre surface. It should also be noted here that the microscopic observation was carried out after the examination of the mechanical properties. The significant flexural tensile strength values of the fabricated TRC are mainly attributable to the textile reinforcement mesh used, which shows excellent anchorage in the matrix due to the perpendicular arrangement of the fibres. In general, there were some isolated fractures of the mesh during the bending test, while most of the deformation of the composite resulted from the matrix-crushing process in the area above the neutral axis of the specimen. The high tensile strength of the reinforcement mesh is attributed to the appropriately arranged glass microfibres visible in Figure 10b. Figure 11a,b show the post-tensile TRC surface formed after the flexural tensile strength at the bending test of the TR-RPC plate. In both cases, the visible lightweight aggregate was destroyed cohesively, indicating the excellent adhesion of the C-S-H phase to its surface. Figure 11a shows, at the same time, the very compact structure of the hydrated calcium silicates resulting from the presence of the pozzolanic additive, i.e., silica fume dosed in an amount equalling as much as 20% of the cement by weight, which directly influences the level of TR-RPC water tightness confirmed in the study.

#### 3.2.3. Water Permeability of TRC

A very important parameter, especially when using a TRC composite as a canoe shell, is the permeability of the composite to water. Water permeability tests were carried out after 7, 28, and 90 days of curing. The results of the tests are given in Table 11.

For the water permeability test, TRC was used as the reference material (REF); in its composition, the lightweight aggregate (see Table 6) was completely replaced with a non-porous quartz aggregate. The aggregate replacement was performed volumetrically. It was measured after 90 days of curing.

The results indicate that the water permeability of the TRC composite is not only due to the porous aggregate but also to the matrix, which seals as the binder hydration progresses. Furthermore, in the case of the reference material, the porosity structure of the aggregate appears to be so dense that at an initial pressure of 1 bar water fills the available composite structure, while further water penetration is practically not observed. The TRC material produced with the lightweight aggregate, due to its highly porous structure, has a water permeability two orders of magnitude higher than that of the reference material, approaching the level of ordinary concretes of strength classes C25/30–C30/37 [40]. The tests showed that the value of the q flux is influenced not only by the presence of the porous lightweight aggregate but also by the hydration level of the binder. Between the 7th day and the 28th day of curing, the water permeability decreased by an order of magnitude, while the further progress of hydration up to the 90th day did not bring about any significant changes.

### 3.3. Numerical Analysis of the Canoe Shell

**Figure 12:**
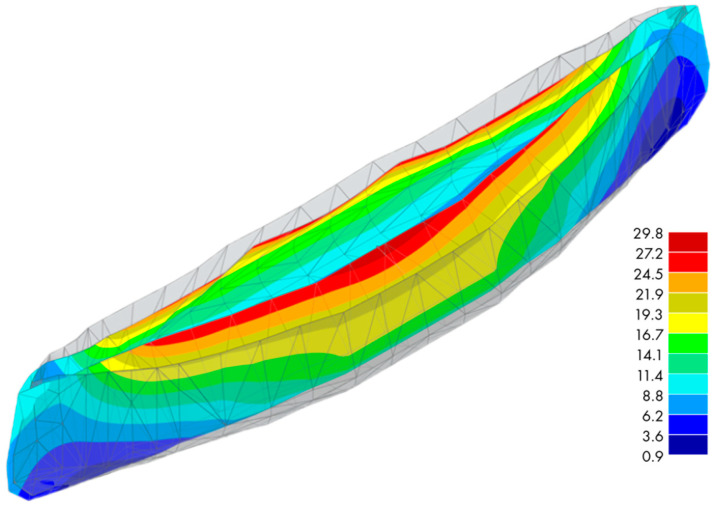
Global deformations of the system, expressed in [mm].

**Figure 13:**
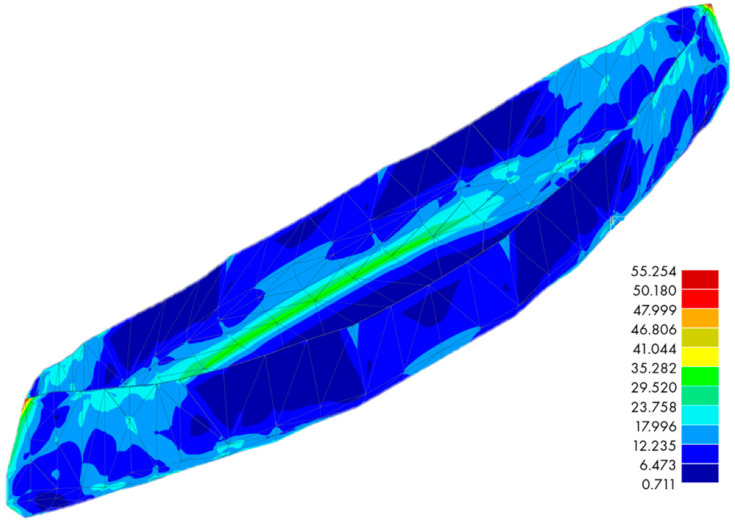
Reduced stresses according to the HMH hypothesis, expressed in [N/mm^2^].

The purpose of the numerical analysis was to verify the deformation of the structure based on the shape adopted by the modelers (analysis of the adopted shape response). The essence was qualitative information related to deformations illustrated in Figure 12 and the concentration of extreme stresses, illustrated in Figure 13.

The analysis was performed using a linear elastic, homogeneous material with specific mechanical parameters. The compressive strength was 60 MPa, and the tensile strength was 12 MPa. A Poisson’s ratio value of 0.2 was adopted [41], while the modulus of elasticity value E = 37 GPa was taken from Eurocode 2 [42]. For simplicity, the shell was modelled with a constant thickness of 4 mm. The RFEM [43] computational system, based on the finite element method of FEM [44], was used to analyse the numerical model. The shell was divided into a mesh of elements with a shape that was close to a square, with a dimension of 10 mm; see Figure 12. This generated a model with a finite number of elements of 55,048. An automatic finite element mesh generator was used during the construction of the model; this was based on the shell entering the calculation system through the standard DXF format, which allowed the transfer of the geometry described through the CAD format. During the analysis, standard four-node or three-node bending elements were used for the quadrilateral and triangular elements, respectively. Mindlin plates were used in the analysis, in which plates with characteristic shear deformations were studied using Timoshenko’s theory. This allows the RFEM to correctly solve problems involving both thick and thin Navier plates.

A shell that mimics the shape of the canoe’s structure was submerged in the liquid up to the side’s edge. By integrating the volume of the boat, its buoyancy was calculated and was about 3 kN. Considering the structure’s weight, it was calculated that the canoe’s buoyancy was around 2.6 kN. As a result, the maximum draft of the boat was set to a value of 35 cm in the calculation model, which was the depth of the shell. The external impact on the shell was simulated as a hydrostatic load, ranging from 0 to 3.5 kN/m^2^ in height. The middle part of the boat adopted an elastic element as a support element and acted as the boundary conditions for both models. As a result of the numerical calculations, the deformation of the shell was obtained, for which the maximum displacement was 29.8 mm.

The next step was to verify the stress state based on the HMH (Huber–Misses–Hencky) hypothesis. Thus, the complex stress state was analysed according to the following Formula (5).

(5) $$\sigma_{red}=\sqrt{\frac{(\sigma_{x}-\sigma_{y}{)}^{2}+(\sigma_{y}-\sigma_{z}{)}^{2}+(\sigma_{z}-\sigma_{x}{)}^{2}}{2}+3(\tau_{xy}^{2}+\tau_{yz}^{2}+\tau_{zx}^{2})}$$

According to the HMH hypothesis, it was shown that the stresses for the coating elements were below the compressive strength of the material; see Figure 13. At the same time, the figure below indicates the places of stress concentration resulting from the resulting notches. A notch effect appears at the junction of the individual coating fragments (places of discontinuity of derivatives at the point for the surface), where an increase in the stress of the coating is evident.

In considering the above analysis during further work, it is necessary to eliminate the notch effect. Numerical analysis based on strain hypotheses allows the optimisation of the shape of the boat in the sense of the shape itself, as well as the amount of embedded material, i.e., the wall thickness.

## 4. Conclusions

The textile-reinforced RPC composite is a material with great application potential. By selecting the right components, it is possible to produce a material with reduced density, high strength, and low water permeability. As a result, it is possible to produce lightweight elements with relatively high strength. Based on the results of this study, the following conclusions and observations can be made:

- The developed composition of the RPC matrix made it possible to achieve a matrix density of 1.23 g/cm^3^ with a total porosity of more than 40%;
- The lightweight matrix allows the achievement of a flexural strength of 12.0 MPa and a compressive strength of 59 MPa. The ratio of compressive strength to flexural strength was as high as 20%, reaching a level four times higher than the ordinary cement matrix.
- The bending tests of the composite (textile-reinforced concrete) plates with the thicknesses of 3 mm and 4 mm showed a behaviour typical for fibre-reinforced textile concretes. Distinct phases of textile-reinforced concrete work can be observed: Phase I—elastic effort of the cement matrix until the first crack; Phase II—load transferred by reinforcement and the formation of cracks; Phase III—cracking of the textile fibres.
- The bending tests of the 1 mm and 2 mm thick composites showed very different behaviours. The materials were flexible and resilient without indicating the three typical phases during the test. The material deflection was more than 4 cm.
- The water permeability results obtained by the GWT method were like those reported in the literature for the ordinary concretes classes C25/30–C30/37. During the first 28 days of curing, the sealing of the matrix structure was visible as an effect of the progressive hydration of the cement. The measured water permeability after 28 days was significantly lower compared to the value recorded after 7 days of curing. It stabilised thereafter. This was confirmed by the obtaining of similar flux “q” values over a longer period.

In summary, this research provides valuable insights into the innovative use of RPC, a lightweight aggregate, and textile and fibre reinforcement in thin-walled cement composites. The successful fabrication of a canoe (PKanoe) using the developed material further supports the proof of concept and demonstrates the practical application and structural performance of the composite in real-world scenarios. The results of this study open perspectives for the exploration of similar materials in various civil engineering applications and highlight the importance of sustainability and tailored material solutions in the evolving field of building materials engineering.

## Figures and Tables

**Table 1:** Physical properties CEM I 52.5.

Feature	Unit	Average Value
Initial setting time	min	180
Water to standard consistency	%	29.2
Volume constancy	mm	0.3
Specific surface	cm^2^/g	4855

**Table 2:** Mechanical properties CEM I 52.5.

Compressive Strength	Unit	Average Value	Requirement
After 2 days	MPa	33.0	30
After 7 days		66.2	52.5

**Table 3:** Chemical properties CEM I 52.5.

Feature	Unit	Average Value
SO_3_	%	2.75
Cr	%	0.058
Loss of ignition	%	2.70
Insoluble residue	%	0.66
Na_2_Oeq	5	0.56

**Table 4:** Characteristics of “Silimic” silica fume.

Chemical Composition	
Component	%wt.
SiO_2_	94.06
Al_2_O_3_	0.74
Fe_2_O_3_	0.78
CaO	0.06
MgO	0.49
Na_2_O_3_	1.43
SO_3_	0.63
Loss of ignition	0.74
**Physical Properties**	
Specific surface [m^2^/g]	22.4
Density [g/cm^3^]	2.23

**Table 5:** Chemical composition of STIKLOPORAS aggregate.

SiO_2_	Al_2_O_3_	K_2_O + Na_2_O	CaO + MgO	Fe_2_O_3_	Other
71.0–73.0	1.5–2.0	13.0–14.0	8.0–10.5	<0.3	<0.5

**Table 6:** Properties of STIKLOPORAS aggregate.

Feature	Unit	0.1–0.3 mm	0.25–0.5 mm
Bulk density	kg/m^3^	400	340
Thermal conductivity	W/mK	-	0.0767
Water absorption	%	18	20
pH	-	10	10
Melting temperature	°C	700	700

**Table 7:** Basic parameters of the glass fibre mesh and the Cem-Fil AR Anticrack HD fibre (data from the producer).

Technical Specification of the Reinforcement Elements		
**Reinforcement Mesh**		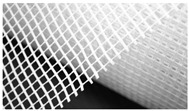
Mesh size (mm)	10 × 10	
Basis weight (g/m^2^)	110	
Tearing strength (N/mm)	40	
**Fibres**		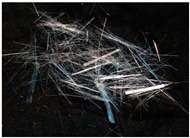
Length (mm)	3	
Filament diameter (μm)	14	
Elastic modulus (GPa)	72	
Pull force (MPa)	1700	
Density (g/cm^3^)	2.68	

**Table 8:** Composition of the RPC mix.

Component	Density (g/cm^3^)	in kg Per m^3^
Cement CEM I 52.5	3.1	899
Silica fume	2.23	180
Lightweight aggregate 0.1–0.3 mm	0.95	111
Lightweight aggregate 0.25–0.5 mm	0.70	192
Water	1.00	216
Glass fibres	2.68	13

**Table 9:** Physical properties of hardened concrete.

Bulk Density (g/cm^3^)	Specific Density (g/cm^3^)	Porosity (%)
1.23 ± 0.03	2.08 ± 0.04	41 ± 2

**Table 10:** Parameters of mercury intrusion porosimetry for RPC matrix.

Bulk Density [g/cm^3^]	Total Porosity [cm^3^/cm^3^]	The Share of Porosity in a Particular Range of Pore Diameters [μm]									
		<0.02	0.02–0.04	0.04–0.08	0.08–0.1	0.1–0.5	0.5–1.0	1.0–10	10–50	50–100	≥100
1.5287	0.369	0.156	0.278	0.189	0.059	0.116	0.000	0.113	0.024	0.012	0.053

**Table 11:** Water permeability according to GWT.

Time of Curing [Days]	REF	7	28	90
q [m/s]	2.4 × 10^−9^	19.8 × 10^−7^	2.1 × 10^−7^	1.4 × 10^−7^
